# Identification and Characterization of Bifunctional Drimenol Synthases of Marine Bacterial Origin

**DOI:** 10.1021/acschembio.2c00163

**Published:** 2022-04-21

**Authors:** Nhu Ngoc Quynh Vo, Yuhta Nomura, Kiyomi Kinugasa, Hiroshi Takagi, Shunji Takahashi

**Affiliations:** † Natural Product Biosynthesis Research Unit, RIKEN Center for Sustainable Resource Science, 2-1 Hirosawa, Wako, Saitama 351-0198, Japan; ‡ Biomolecular Characterization Unit, RIKEN Center for Sustainable Resource Science, 2-1 Hirosawa, Wako, Saitama 351-0198, Japan; § Graduate School of Science and Engineering, Saitama University, 255 Shimo-Okubo, Sakura-ku, Saitama 338-8570, Japan

## Abstract

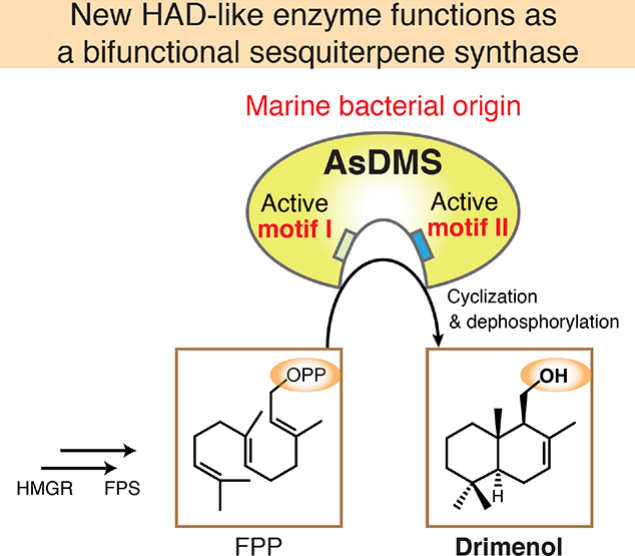

Natural drimane-type sesquiterpenes, including drimenol, display diverse biological activities. These active compounds are distributed in plants and fungi; however, their accumulation in bacteria remains unknown. Consequently, bacterial drimane-type sesquiterpene synthases remain to be characterized. Here, we report five drimenol synthases (DMSs) of marine bacterial origin, all belonging to the haloacid dehalogenase (HAD)-like hydrolase superfamily with the conserved DDxxE motif typical of class I terpene synthases and the DxDTT motif found in class II diterpene synthases. They catalyze two continuous reactions: the cyclization of farnesyl pyrophosphate (FPP) into drimenyl pyrophosphate and dephosphorylation of drimenyl pyrophosphate into drimenol. Protein structure modeling of the characterized *Aquimarina spongiae* DMS (AsDMS) suggests that the FPP substrate is located within the interdomain created by the DDxxE motif of N-domain and DxDTT motif of C-domain. Biochemical analysis revealed two aspartate residues of the DDxxE motif that might contribute to the capture of the pyrophosphate moiety of FPP inside the catalytic site of AsDMS, which is essential for efficient cyclization and subsequent dephosphorylation reactions. The middle aspartate residue of the DxDTT motif is also critical for cyclization. Thus, AsDMS utilizes both motifs in the reactions. Remarkably, the unique protein architecture of AsDMS, which is characterized by the fusion of a HAD-like domain (N-domain) and a terpene synthase β domain (C-domain), significantly differentiates this new enzyme. Our findings of the first examples of bacterial DMSs suggest the biosynthesis of drimane sesquiterpenes in bacteria and shed light on the divergence of the structures and functions of terpene synthases.

Terpenoids, as the most diverse class of natural products, are distributed in all living taxa, including plants, fungi, bacteria, and protists. Terpenoids are well-known for their wide range of biological and pharmacological activities.^1−3^ Terpene synthases are key biosynthetic determinants of terpenoid chemodiversity; they fold the universal prenyl diphosphate precursors, including geranyl (C_10_), farnesyl (C_15_), and geranylgeranyl (C_20_) diphosphates, followed by carbocation formation to produce a range of scaffolds promoting structural and stereochemical diversity.^4−6^ The two distinct classes of terpene synthases use different substrate activation mechanisms. Class I enzymes initiate reactions through ionization of a pyrophosphorylated substrate,^7^ whereas class II enzymes initiate reactions through protonation of carbon–carbon double bonds.^8^ Both mechanisms produce carbocation intermediates that undergo a carbocation cascade to generate various terpenoids. Comparison of terpene synthases reveals that the protein architecture is modular in nature and can contain up to three domains, termed α, β, and γ.^9−11^ Single-domain enzymes are currently described for only the α-domain microbial class I terpene synthases, while other class I enzymes exhibit αβ or αβγ architectures. The class II terpene synthases feature βγ or αβγ architectures. Recently, a single β-domain class II terpene synthase has been noted for the merosterolic acid synthase,^12^ underlining the diversity of terpene synthase structures.

Sesquiterpene synthases (STSs) catalyze the biosynthesis of structurally diverse sesquiterpenes, a family of C_15_ terpenoids, from farnesyl pyrophosphate (FPP).^13^ The overall α- or αβ-domain architecture, with the active site in the α-domain, serves as a template for catalysis by an STS. This ensures that the substrate FPP and subsequent intermediates adopt only those conformations resulting in the formation of the correct product(s).^10^ A large number of STSs have been characterized in plants^14,15^ and fungi;^16^ however, much less is known about STSs of bacterial origin, given the relatively small number of characterized bacterial sesquiterpenes.^17^ Indeed, drimanes, one of the unique sesquiterpenes displaying antimicrobial, antifeedant, and insecticidal effects,^18^ are known for their predominant distribution in plants^19^ and fungi;^20^ however, the extent of accumulation of these active compounds in bacteria remains unknown. Consequently, no reports exist on bacterial drimane-type STSs, which are key biosynthetic enzymes of drimanes. Drimenol serves as the central biosynthetic precursor of various naturally occurring drimane sesquiterpenes,^19^ and it is a strong broad-spectrum antifungal agent.^21^ To date, two drimenol synthases (DMSs) of plant origin have been identified in valerian (*Valeriana officinalis*)^22^ and water pepper (*Persicaria hydropiper*).^23^ Therefore, it is important to discover new bacterial drimane STSs, including DMSs to identify novel sesquiterpenes in bacteria.

Here, we report the functional characterization of five DMSs of marine bacterial origin, all of which catalyze the biosynthesis of drimenol from FPP. The DMS enzymes contain two universally conserved aspartate-rich motifs of class I and II terpene synthases, which are rare for STSs. Using protein structure modeling and site-directed mutagenesis, *Aquimarina spongiae* DMS (AsDMS) enabled the characterization of the catalytic mechanism of drimenol biosynthesis and suggested a unique domain architecture of this new enzyme.

## Results and Discussion

### Genomic Data Mining to Discover Bacterial DMSs

**Figure 1:**
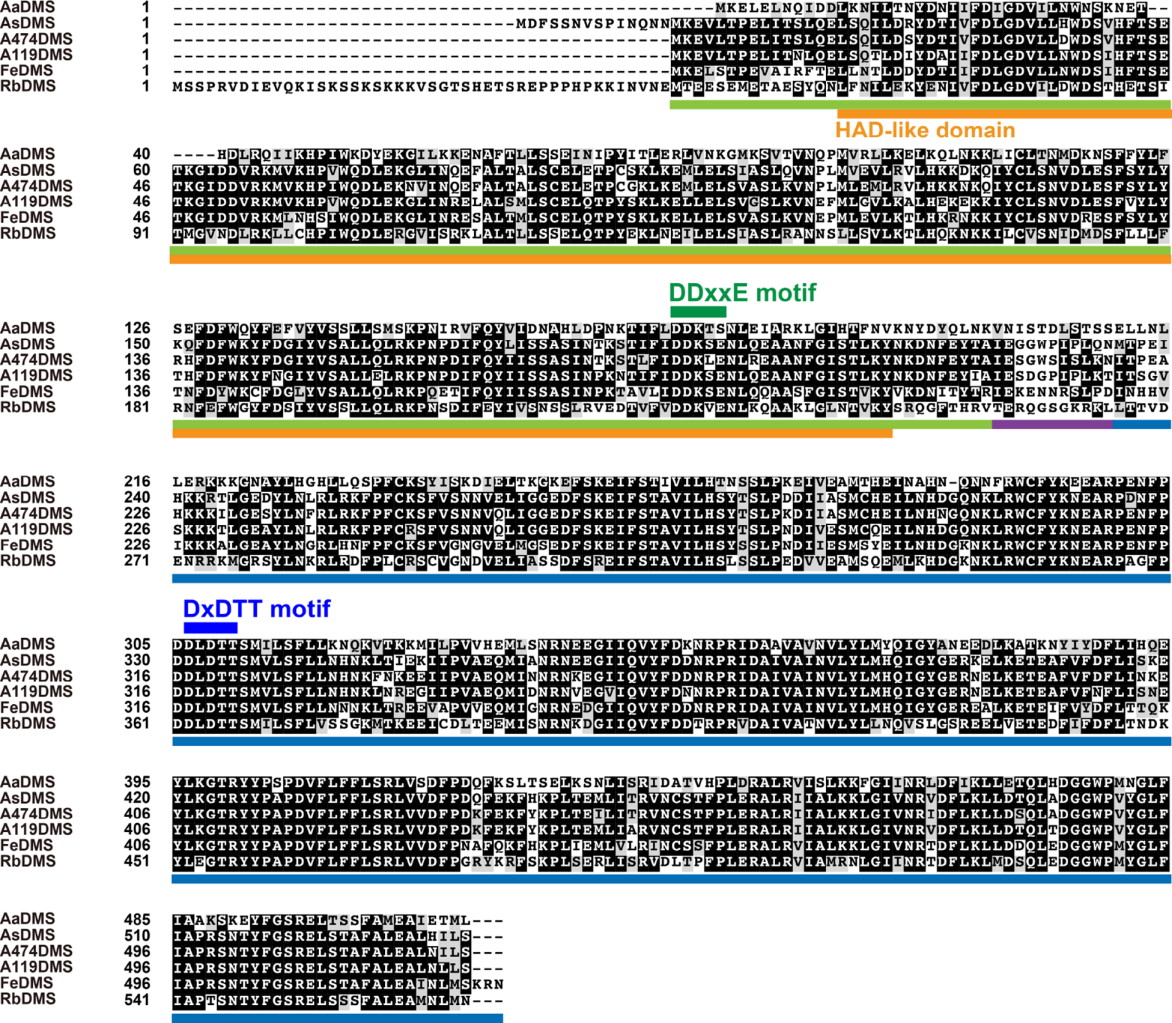
Multiple sequence alignment of six bacterial DMS candidate proteins. The species names are abbreviated as follows: Aa, *A. aggregate*; As, *A. spongiae*; A474, *Aquimarina* sp. AU474; A119, *Aquimarina* sp. AU119; Fe, *F. eckloniae*; and Rb, *Rhodobacteraceae* KLH11. The protein sequences were aligned and colored using the GenomeNet ClustalW 1.83 (https://www.genome.jp/tools-bin/clustalw) and BoxShade 3.21 servers (https://embnet.vital-it.ch/software/BOX_form.html). The N- and C-domains, and their linker region, are underlined in green, blue, and purple, respectively. The HAD-like domain is underlined in orange. The conserved aspartate-rich motifs DDxxE_195–199_ and DxDTT_331–335_ are indicated in dark green and dark blue, respectively.

The previously reported *AstC*, a fungal drimane STS gene from *Aspergillus oryzae*, belongs to a haloacid dehalogenase (HAD)-like hydrolase superfamily.^20^ Therefore, we searched a bacterial protein database^24^ for AstC homologues using a hidden Markov model (HMM).^25^ This analysis provided 1252 candidate sequences from bacteria; the candidates were then subjected to multiple sequence alignments. It was reported that AstC conserves a typical DxDTT motif found in class II diterpene synthases. Accordingly, we analyzed the 1252 candidate sequences and extracted only those containing the DxDTT motif. Finally, four sequences were retrieved from bacterial clades as putative candidates. We then searched the National Center for Biotechnology Information (NCBI) database using the Basic Local Alignment Search Tool (BLAST), with the four candidate sequences as queries, and found two additional homologues from different bacteria. Six putative DMSs were obtained from *Aquimarina aggregate* (AaDMS), *A. spongiae* (AsDMS), *Aquimarina* sp. AU474 (A474DMS), *Aquimarina* sp. AU119 (A119DMS), *Flavivirga eckloniae* (FeDMS), and *Rhodobacteraceae* KLH11 (RbDMS) (Figure 1). The six protein sequences are available in NCBI under the accession numbers: WP_066312843 (AaDMS), WP_084549426 (AsDMS), WP_109300389 (A474DMS), WP_109437248 (A119DMS), WP_102757879 (FeDMS), and WP_008759207 (RbDMS).

### Functional Analysis of Bacterial DMS Candidates

**Figure 2:**
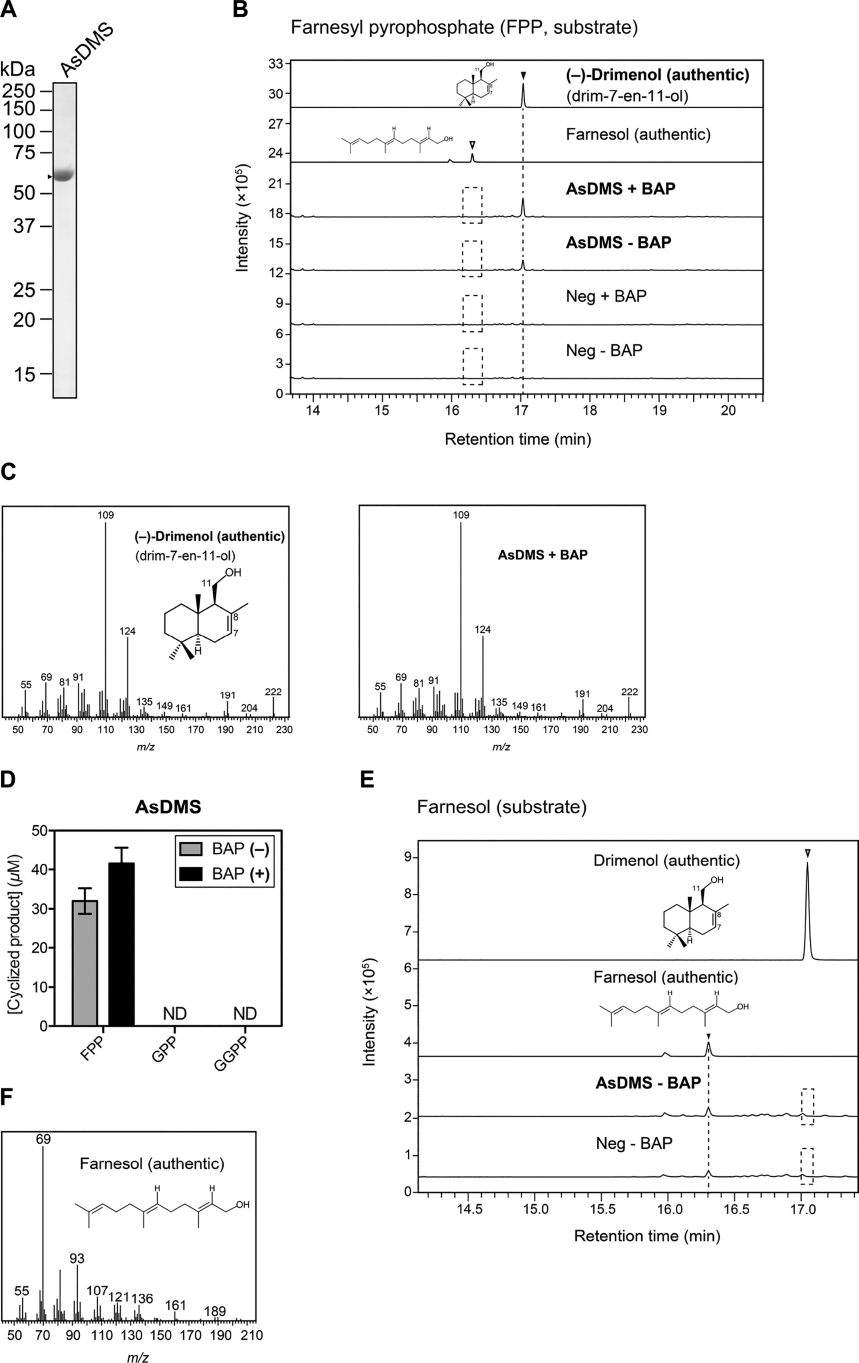
Catalytic activity of the recombinant AsDMS protein. (A) Sodium dodecyl sulfate-polyacrylamide gel electrophoresis (SDS-PAGE) analysis of purified AsDMS protein from *E. coli*. The gel was stained with Coomassie Brilliant Blue, and the positions of molecular markers are indicated. The *arrowhead* indicates the AsDMS band. (B) GC–MS chromatograms of the authentic (−)-drimenol (drim-7-en-11-ol) and farnesol compounds and *in vitro* reaction products. Enzymatic assays of the purified AsDMS protein were performed individually in the presence of FPP as a substrate, with or without alkaline phosphatase (BAP) treatment. The reactions in the absence of protein (Neg) were used as negative controls. (C) Mass spectra of the authentic (−)-drimenol and the reaction product detected in (B). (D) Comparison of substrate selectivity of the AsDMS protein. The enzyme reactions were performed individually under the same conditions as in (B) except for the inclusion of additional substrates: GPP, geranyl pyrophosphate, and GGPP, geranylgeranyl pyrophosphate. Data are the means ± SD of three independent experiments performed with the same preparation of purified protein. ND, not detectable. (E) GC–MS analyses of the authentic drimenol and farnesol and *in vitro* enzyme reaction of the recombinant AsDMS protein with farnesol as a substrate. The reaction was performed without BAP. (F) Mass spectrum of the authentic farnesol. The GC–MS data in (B,C) and (E,F) represent three independent reactions performed with the same preparation of purified protein. Dashed boxes indicate that no desired reaction products were detected. As, *A. spongiae* and *m*/*z*, mass-to-charge ratio.

To assess the catalytic function of the six DMS candidates, we expressed the recombinant proteins fused with the N-terminal octa-histidine-tag (His_8_-tag) in *Escherichia coli*. The three recombinant proteins (AaDMS, AsDMS, and RbDMS) were visibly expressed in soluble fractions (Figure S1), and these soluble proteins were further isolated from the cells by cobalt-immobilized metal affinity chromatography (Figures 2A, S3A, and S4A). Although the expression of the remaining three candidates (A474DMS, A119DMS, and FeDMS) was mainly observed in the pellet (Figure S1), we attempted to isolate them from the supernatant and successfully obtained purified proteins albeit their low protein amounts (Figure S3A).

**Figure 3:**
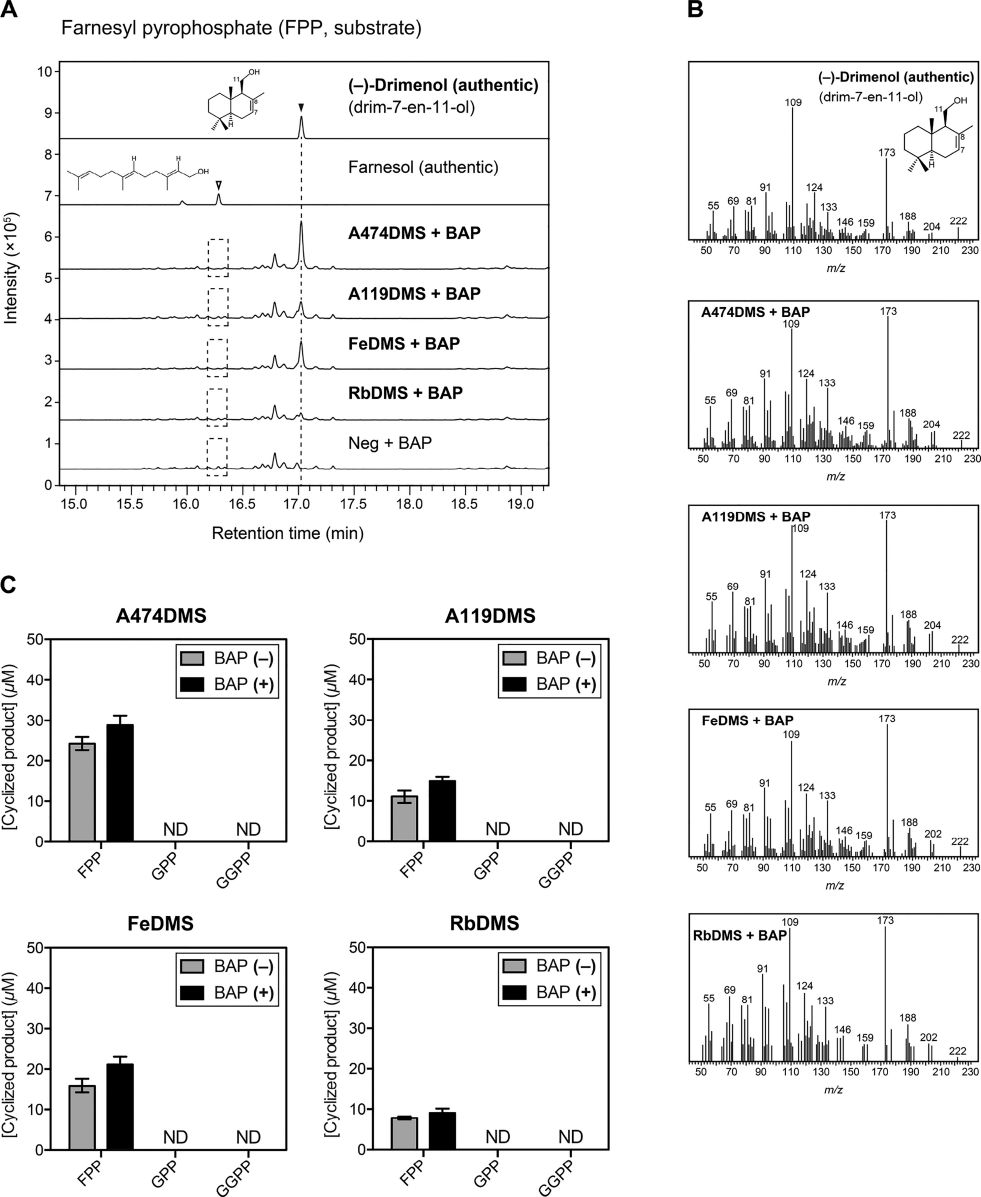
Catalytic activity of the four recombinant proteins A474DMS, A119DMS, FeDMS, and RbDMS. (A) GC–MS chromatograms of the authentic (−)-drimenol (drim-7-en-11-ol) and farnesol compounds and *in vitro* reaction products. The enzymatic assays of each purified protein were performed individually in the presence of FPP as a substrate, with or without alkaline phosphatase (BAP) treatment. The reactions in the absence of protein (Neg) were used as negative controls. (B) Mass spectra of the authentic (−)-drimenol and each reaction product detected in (A). The GC–MS data in (A,B) represent three independent reactions performed with the same preparation of each purified protein. Dashed boxes indicate that no desired reaction products were detected. (C) Comparison of the substrate selectivity of A474DMS, A119DMS, FeDMS, and RbDMS proteins. The enzyme reactions of each protein were performed individually under the same conditions as in (A), except for the inclusion of additional substrates: GPP, geranyl pyrophosphate, and GGPP, geranylgeranyl pyrophosphate. Data are the means ± SD of three independent experiments performed with the same preparation of each purified protein. ND, not detectable; A474, *Aquimarina* sp. AU474; A119, *Aquimarina* sp. AU119; Fe, *F. eckloniae*; Rb, *Rhodobacteraceae* KLH11; and *m*/*z*, mass-to-charge ratio.

**Figure 4:**
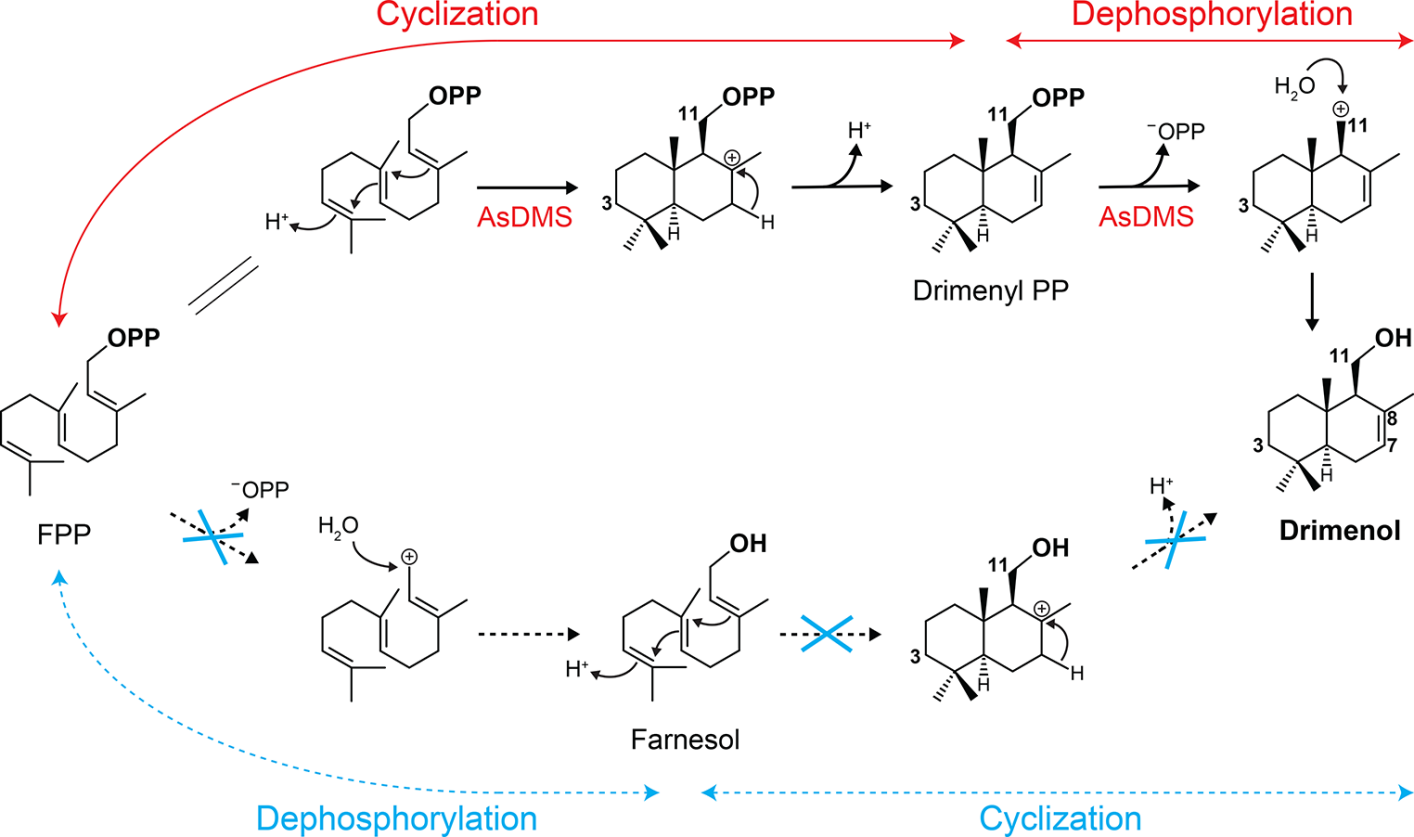
Proposed mechanism of drimenol biosynthesis catalyzed by the bifunctional AsDMS. Protonation at C3 mediated by the DxDTT motif occurs first, followed by double-bond rearrangements and deprotonation to produce drimenyl pyrophosphate (the cyclization step). Next, cleavage of the diphosphate group by the DDxxE motif and Mg^2+^ ions generates carbocation, which could be quenched by a water molecule to produce drimenol (the dephosphorylation step).

To examine whether the six candidate proteins had DMS activity, we performed *in vitro* enzymatic activity assays in the presence of FPP as a substrate. The reaction mixtures were treated with or without alkaline phosphatase (BAP) to check whether these six candidates could dephosphorylate FPP or the reaction products. The products were analyzed by gas chromatography–mass spectrometry (GC–MS). As a result, the reactions of five proteins (AsDMS, A474DMS, A119DMS, FeDMS, and RbDMS) with FPP yielded a single compound corresponding to the authentic (−)-drimenol (drim-7-en-11-ol), which is a sesquiterpene alcohol, in terms of the retention time (17.047 min) and exact mass fragmentation pattern (parent mass *m*/*z* = 222) (Figures 2B,C and 3A,B). This suggests that these proteins function as DMSs catalyzing the biosynthesis of (−)-drimenol from FPP. The (−)-drimenol was detected in the reactions without BAP treatment, indicating dephosphorylation activity of these enzymes; this is dissimilar with AstC, which only catalyzes the cyclization of FPP into an isomeric drimenyl pyrophosphate and lacks dephosphorylation activity.^20^ We deduce that, in the absence of BAP, AsDMS catalyzed drimenol synthesis through both cyclization and dephosphorylation activities; however, there might be cyclic diphosphate intermediates, such as drimenyl pyrophosphates, which may not have undergone the dephosphorylation step. Hence, the addition of BAP seemed to favor dephosphorylation and enhance the DMS activity. In fact, the addition of BAP enhanced drimenol production by the five DMSs (Figures 2D and 3C). We did not detect farnesol, another possible precursor of (−)-drimenol (Figure 4), in the reactions, even in the presence of BAP which is assumed to dephosphorylate FPP to produce farnesol (Figures 2B and 3A). Using AsDMS, we further investigated if farnesol could serve as an intermediate during the catalysis. However, when farnesol was used as a substrate, no drimenol was obtained (Figure 2E,F). These findings suggest that farnesol was not an intermediate in drimenol biosynthesis by AsDMS (Figure 4). In addition, the remaining candidate AaDMS could not synthesize sesquiterpenes, including drimenol, when using FPP as the substrate (Figure S4B).

Next, to check whether the five DMSs have high specificities for FPP as a substrate, we performed the enzymatic reactions of these proteins using geranyl pyrophosphate (GPP) or geranylgeranyl pyrophosphate (GGPP) as substrates. No reaction products were detected in the *in vitro* reactions (Figures 2D and 3C), suggesting that the AsDMS, A474DMS, A119DMS, FeDMS, and RbDMS enzymes function as specific STSs. With the activity of BAP, we only detected geraniol as the precursor of monoterpenes, in the presence of GPP as the substrate (Figures S2A–B and S3B–C); however, geranyl geraniol, the precursor of diterpenes, was not observed with the GGPP substrate (Figures S2C–D and S3D). Similar observations were noted for AaDMS (Figure S4C–E) due to the BAP activity.

To the best of our knowledge, this is the first report on drimane STSs of bacterial origin, which catalyze the cyclization of FPP into (−)-drimenol. We further characterized AsDMS that was highly expressed in the supernatant. The Michaelis–Menten kinetic parameters were calculated by quantifying the amount of the drimenol product using GC–MS. A saturation curve was obtained from the Michaelis–Menten curve of the AsDMS protein (Figure S5). The half-maximal saturation concentration (Michaelis constant, *K*_m_) for FPP, turnover number (*k*_cat_), and *k*_cat_/*K*_m_ values were 9.59 ± 2.15 μM, 0.086 ± 0.01 s^–1^, and 0.009 ± 0.001 s^–1^ μM^–1^, respectively. This result indicates that *AsDMS* encodes a catalytically active STS, and its kinetic properties agree with those reported for known bacterial STSs.^26−28^ Notably, the catalytic efficiency of AsDMS is remarkably higher than that of plant-derived *V. officinalis* DMS, with both enzymes having similar *K*_m_ values.^22^ This might result from the fact that plant STSs involved in secondary metabolism have low *k*_cat_/*K*_m_ values in the range of 0.001 s^–1^ μM^–1^ or even lower.^29^

### Structural Insights into the Catalytic Active Site of AsDMS

To obtain useful insights into the structural basis of AsDMS protein, we constructed a homology model of the AsDMS protein using the automated SWISS-MODEL^30^ pipeline. There were no ideal template proteins to construct a full-length model of AsDMS. However, when we predicted the models of the N- and C-domains of AsDMS separately, we identified suitable template structures, implying a multidomain architecture of this enzyme, which was also predicted by AlphaFold2^31^ (data not shown). The crystal structures of the HAD-like phosphatase YihX from *E. coli* (PDB ID: 2B0C chain A)^32^ and *ent*-copalyl diphosphate synthase PtmT2 from *Streptomyces platensis* (PDB ID: 5BP8 chain A)^33^ were suitable template structures for homology modeling of the N- and C-domains, respectively. We next assembled these domain models into the entire AsDMS model using domain-enhanced modeling (DEMO)^34^ and predicted the spatial arrangements of the active site-defining residues within the AsDMS structure.

**Figure 5:**
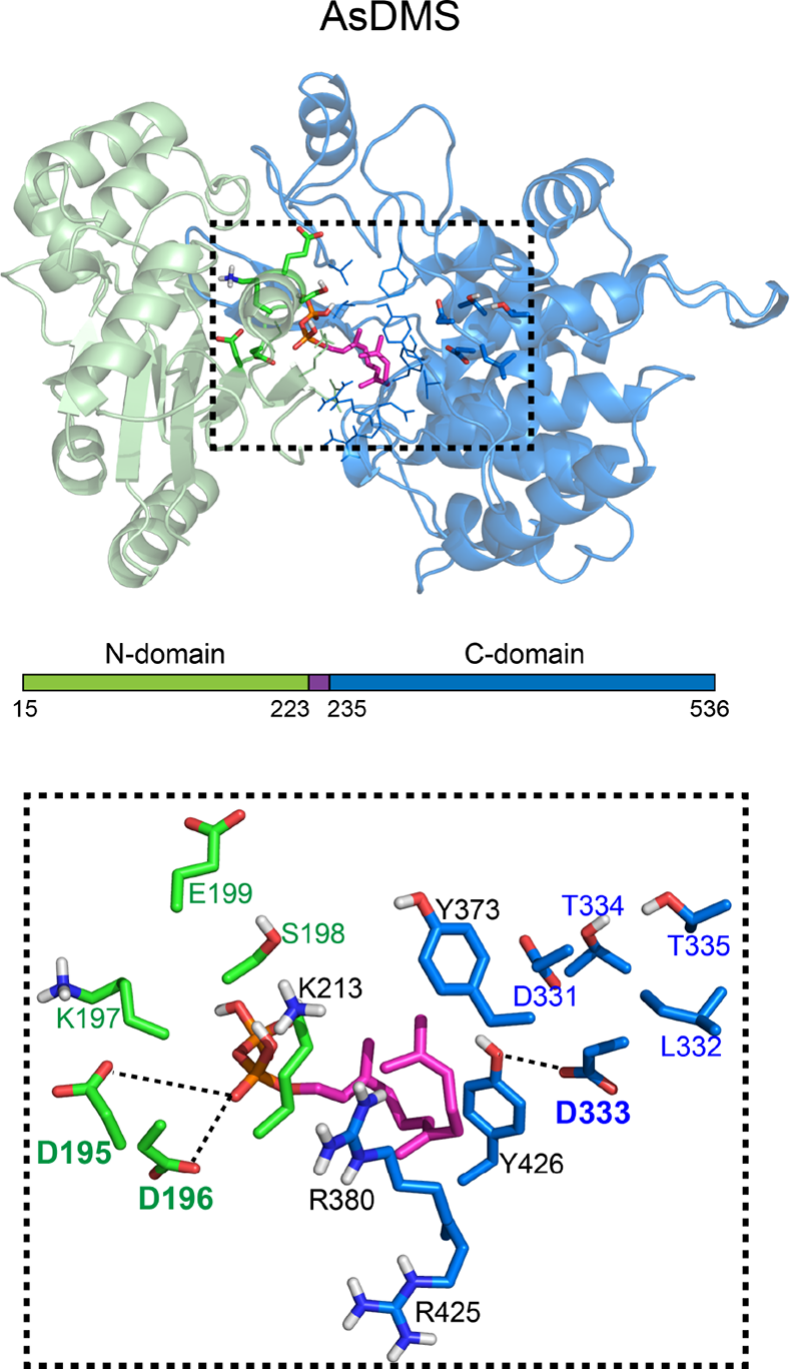
Virtual docking of the farnesyl diphosphate (FPP) substrate onto the homology model of the AsDMS protein. The upper panel shows an overview map of the structural model (N-domain, pale green and C-domain, pale blue) of the AsDMS protein docked with FPP (magenta stick rendering; red, oxygen). FPP is positioned within the interdomain shared by both the N- and C-domains, which is shown in the black-dotted box. The lower panels show a close-up view of the predicted spatial arrangements of the amino acids lining the substrate-binding cavity in the AsDMS protein. The side chains of residues located at the N-domain are shown in atomic coloring (green, carbon and red, oxygen), while those located at the C-domain are displayed in blue for carbon and red for oxygen. The conserved DDxxE_195–199_ (N-domain) and DxDTT_331–335_ (C-domain) motifs are shown in dark green and dark blue font, respectively, with the catalytically essential amino acids (D195, D196, and D333) in bold font. Other interacting residues (K213, Y373, R380, R425, and Y426) are in black font. Possible hydrogen bonds are indicated by black-dotted lines. As, *A. spongiae*.

As depicted in Figure 5 (upper panel), the DDxxE_195–199_ motif at the N-domain and DxDTT_331–335_ motif (a variation of the DxDD motif)^20,35,36^ at the C-domain were positioned inside the AsDMS model. These motifs are highly conserved among class I and II terpene synthase enzymes, respectively. It is interesting to note that a large cavity pocket exists between the N- and C-domains. We therefore speculate that this pocket is a substrate-binding site for FPP. To further investigate the spatial position of the substrate-binding pocket in AsDMS, virtual docking of FPP onto the AsDMS model was performed by AutoDock Vina.^37^ The docked structures were analyzed, and enzyme–ligand interactions were observed. The results demonstrated that the FPP substrate fits within the interdomain shared by the DDxxE_195–199_ and DxDTT_331–335_ motifs (Figure 5, lower panel). Analysis of the residues in the substrate-binding site suggested that the key D195 and D196 residues, as the first and second aspartate, respectively, of the DDxxE_195–199_ motif form hydrogen bonds with the pyrophosphate moiety of FPP, indicating that these aspartate residues play a role in positioning FPP in the catalytic site. Also, the basic K197 residue of the DDxxE_195–199_ motif was predicted to be located adjacent to the D195/D196 cluster in the AsDMS model.

In addition, the model indicated the position of the DxDTT_331–335_ motif, especially the middle aspartate residue D333, which was near the FPP substrate-binding pocket. D333 was predicted to point toward the farnesyl moiety of FPP and showed possible interactions with the binding-site residue Y426. There were also possible interactions between the farnesyl moiety of FPP and residues R380, R425, and Y426.

### Identification of Essential Residues of AsDMS for Drimenol Synthesis

**Figure 6:**
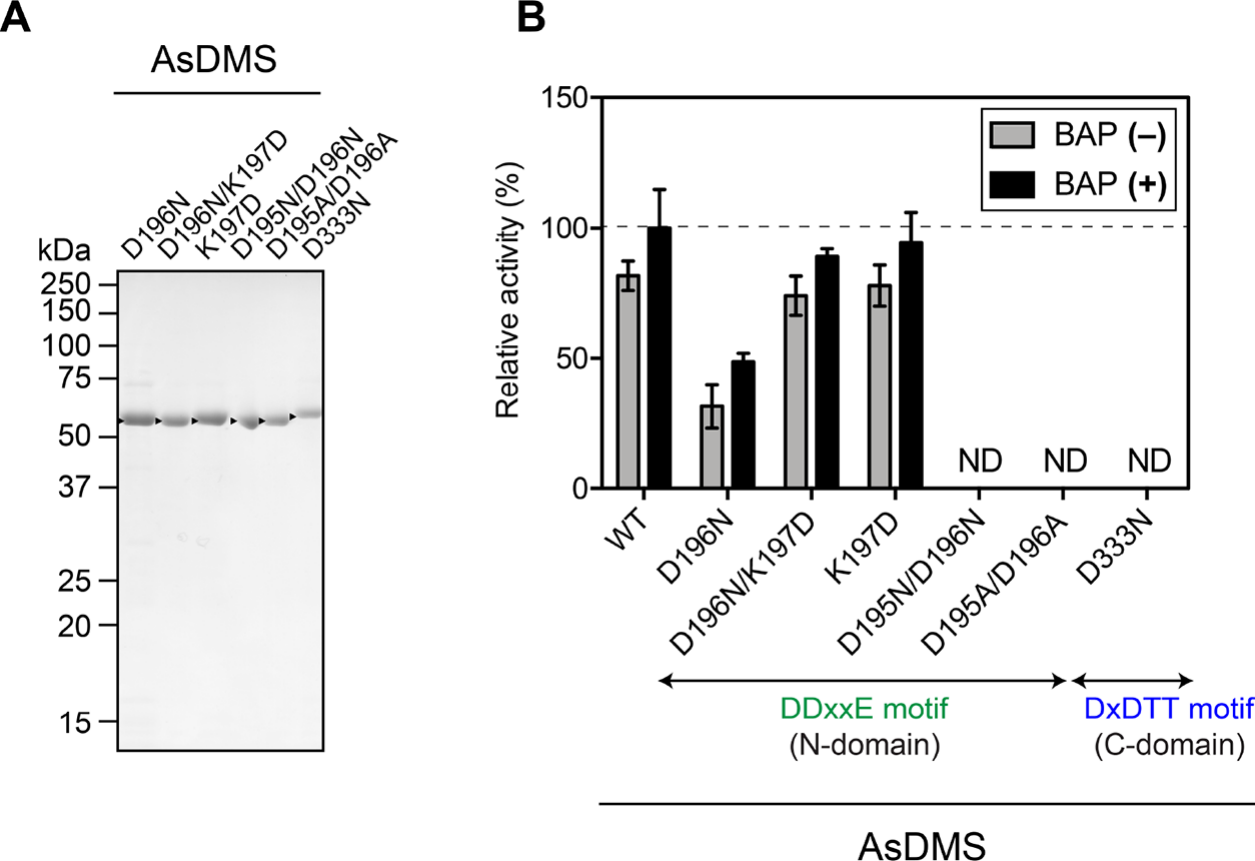
Comparison of catalytic activity among the WT AsDMS protein and its residue-swapped mutant proteins. (A) SDS-PAGE analysis of purified mutant AsDMS proteins from *E. coli*. The gel was stained with Coomassie Brilliant Blue, and the positions of molecular markers are indicated. *Arrowheads* indicate the mutant AsDMS bands. (B) The enzyme reactions of these purified proteins were performed individually under the same conditions, with or without the inclusion of alkaline phosphatase (BAP). The catalytic activity of AsDMS in the presence of BAP was set as 100% (dashed line). Data are expressed as means ± SD of three independent experiments performed with the same preparation of each purified protein. ND, not detectable; As, *A. spongiae*.

The AsDMS model structure suggests putative key catalytic amino acid residues in the active site. Based on the modeling results, we performed site-directed mutagenesis to create a series of AsDMS variants with substitution mutations in the DDxxE_195–199_ motif (D196N; D196N/K197D; K197D; D195N/D196N; and D195A/D196A) and the DxDTT_331–335_ motif (D333N). The recombinant AsDMS mutant proteins were expressed in *E. coli* and further purified (Figure 6A). The reactions of each purified mutant protein with FPP, in the presence or absence of BAP, were analyzed using GC–MS, and the DMS activity of the wild-type (WT) AsDMS under BAP treatment was set as 100% (Figure 6B).

The D196N mutant displayed a significant decrease in activity with respect to the native enzyme by 50% with BAP and 62% without BAP (Figure 6B). However, a double mutant D196N/K197D, which was obtained by adding a negatively charged aspartate residue (K197D) to D196N, increased its drimenol synthesis activity with respect to D196N by 44 and 57% in the presence and absence of BAP, respectively, which is comparable to the activity of the WT (Figure 6B). These results suggest the importance of the negatively charged residues of the DDxxE_195–199_ motif, as observed in the aspartate-rich motifs of class I STSs.^38,39^ Surprisingly, the K197D mutation, which produces a series of three acidic aspartate residues (DDDSE_195–199_) and thus enhances the acidity of the motif, exhibited almost equipotent activity to that of the WT, with and without the inclusion of BAP. This observation indicates that the positive charge of the basic residue K197 plays no role in catalysis by the DDxxE_195–199_ motif. Furthermore, the D195N/D196N and D195A/D196A variants, which were obtained by mutating the two aspartate residues to asparagine and alanine, respectively, entirely lost their DMS activity, although the D196N mutation lacking only the second aspartate did exhibit some activity (Figure 6B). Therefore, we strongly suggest that the first aspartate (D195) and second aspartate (D196) residues are responsible for catalysis by the DDxxE_195–199_ motif, in which these residues could play a key role in positioning FPP in the active site through a hydrogen-bonding network with Mg^2+^ ions and a pyrophosphate group, as proposed by the previously reported STSs.^38,39^ Disruption of the hydrogen-bonding network would result in a loss of enzyme activity.

In terms of the DxDTT_331–335_ motif, the substitution of the middle aspartate residue D333 with asparagine resulted in a complete loss of enzyme activity of producing the cyclized drimenol product (Figure 6B). This suggests that the D333N mutant might decrease the acidity of the DxDTT_331–335_ motif, such that the cyclization reaction did not occur. The D333 residue might donate a proton that attacks the terminal double bond of FPP to initiate cyclization, as suggested by the previously reported class II diterpene synthases.^35,36,40,41^ These results indicate that AsDMS utilizes both the active DDxxE_195–199_ and DxDTT_331–335_ motifs for its activity to catalyze two continuous reactions: the cyclization of FPP into drimenyl pyrophosphate and dephosphorylation of drimenyl pyrophosphate into drimenol.

Consequently, we proposed a biosynthetic mechanism of drimenol by AsDMS in which this bifunctional enzyme may proceed with protonation at C3 by the DxDTT_331–335_ motif, followed by double-bond rearrangements and deprotonation to produce drimenyl pyrophosphate. Next, the cleavage of the diphosphate group by the DDxxE_195–199_ motif and Mg^2+^ ions could generate a carbocation that can be quenched by a water molecule to produce drimenol (Figure 4). Moreover, when catalysis by the DDxxE_195–199_ motif was impaired by the D195N/D196N or D195A/D196A mutations, the cyclized product (drimenol) was not detected despite the addition of BAP to the reaction mixture to compensate for the loss of dephosphorylation activity. This result indicates a complete loss of cyclization activity; therefore, we conclude that the N- and C-domains of AsDMS share a single active site for sequential cyclization and dephosphorylation reactions (Figure 5), which differs from the bifunctional class I–II abietadiene synthase that encodes two distinct active sites in a single polypeptide.^42^

### Unique Architectural Assembly and the Evolution of Two Domains of AsDMS

AsDMS is characterized by an N-terminal domain that involves a HAD-like domain (residues 30-214) comprising a DDxxE_195–199_ motif found in class I terpene synthases and a C-terminal domain that comprises a DxDTT_331–335_ motif found in class II diterpene synthases. Therefore, we questioned whether the assembly of a HAD-like domain (N-domain) and a terpene synthase domain (C-domain) was the new evolutionary target of the HAD superfamily and terpene synthase enzymes.

**Figure 7:**
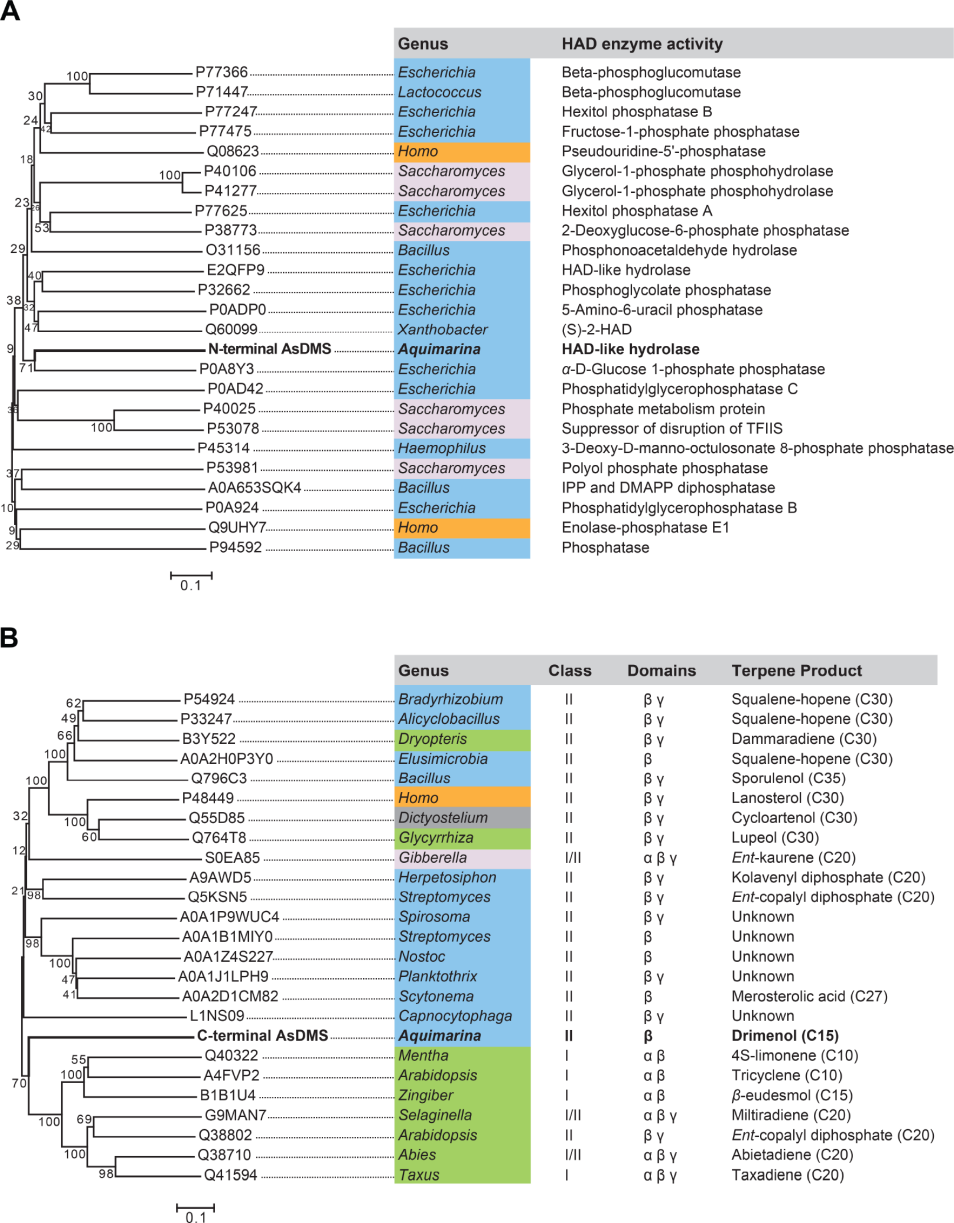
Neighbor-joining phylogenetic trees of (A) the N-terminal domain of AsDMS (bold black font) with functionally characterized HAD-like hydrolases and (B) the C-terminal domain of AsDMS (bold black font) with selected β-domains from functionally characterized and unknown terpene synthases. The bar indicates 0.1 amino acid substitution per site. The *numbers* shown next to branches are the percentages of replicate trees in which associated taxa clustered together in bootstrap tests with 1000 replicates. Organisms are colored according to the phylogeny of their origin: bacteria—blue, fungi—light gray, plants—green, protists—gray, and animals—orange. The accession number, HAD enzyme activity, terpene synthase class, domain architecture, and terpene product are indicated. As, *A. spongiae*.

**Figure 8:**
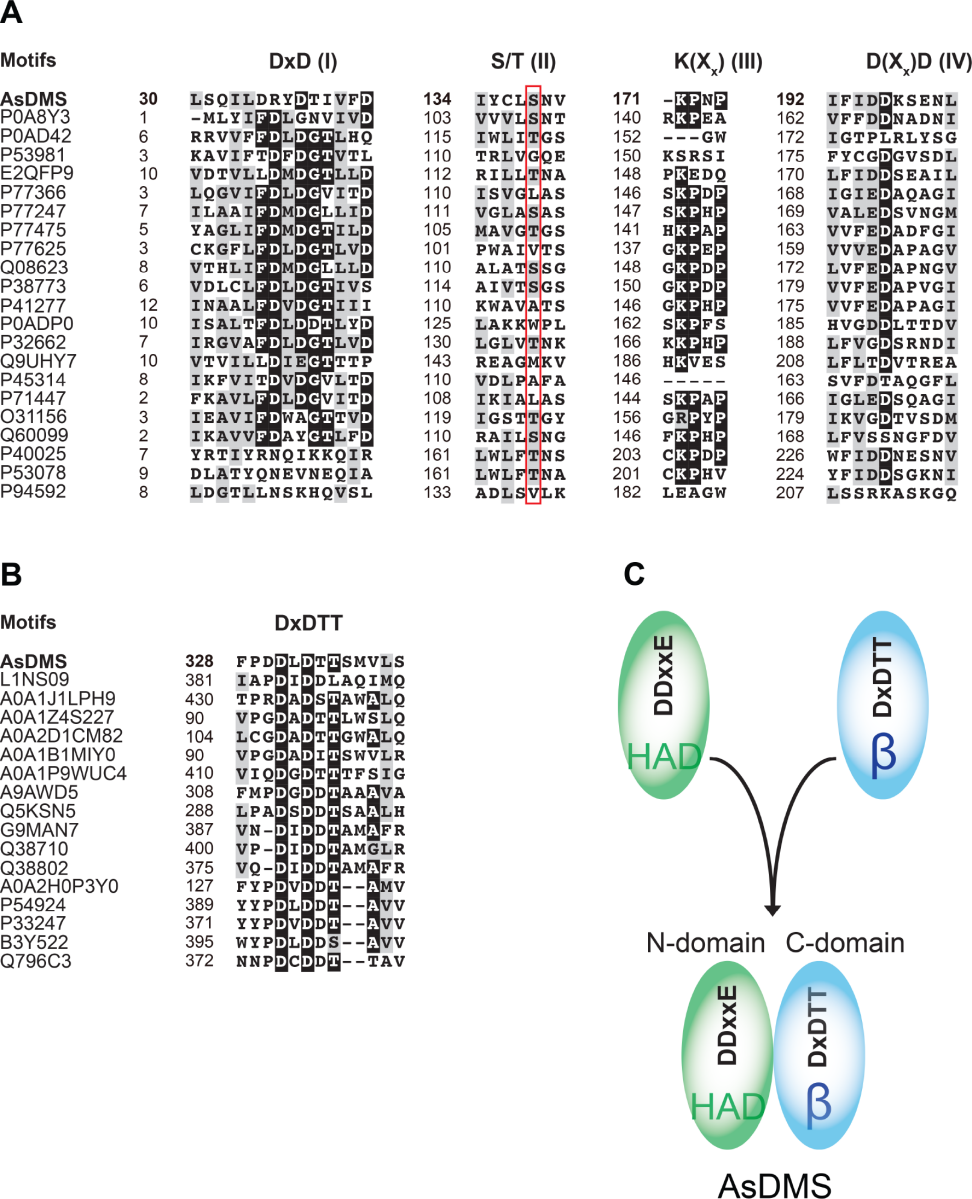
Unique architectural assembly and evolution of two domains of AsDMS. (A) Sequence comparisons of the N-domain of AsDMS with selected HAD-like hydrolases show the presence of four short conserved HAD signature motifs: DxD (motif I), S/T (motif II), K(X_x_) (motif III), and D(X_x_)D (motif IV). A red box denotes the position of the S/T motif. (B) Sequence comparisons of the C-domain of AsDMS with selected β domains of terpene synthases indicate the presence of the DxDTT motif of AsDMS, which corresponds closely to the DxDD or DxDTT motifs of terpene synthases. The protein sequences were aligned and colored using the GenomeNet ClustalW 1.83 (https://www.genome.jp/tools-bin/clustalw) and BoxShade 3.21 servers (https://embnet.vital-it.ch/software/BOX_form.html). (C) The unique architecture of AsDMS is characterized by the fusion of a HAD-like domain (N-domain) comprising a DDxxE motif with a terpene synthase β domain (C-domain) comprising a DxDTT motif. As, *A. spongiae*.

We traced the phylogenetic relationship of the N-terminal domain of the AsDMS enzyme with functionally characterized HAD-like hydrolases. Notably, the N-terminal AsDMS clustered with known bacterial HAD phosphatases and was homologous to a phosphatase enzyme from *E. coli* (accession: P0A8Y3)^32^ (Figure 7A). It is proposed that HAD phosphatases are dominant enzymes of the HAD-like hydrolase superfamily, which is one of the largest enzyme superfamilies found in all organisms.^43^ The HAD phosphatases conduct catalysis using an aspartate residue for nucleophilic attack, which requires Mg^2+^ as an essential cofactor. Extensive sequence comparisons of the N-domain of AsDMS with HAD-like hydrolases showed the presence of four short conserved HAD signature motifs^44,45^ (Figure 8A). The N-terminal motif I comprises the aspartate nucleophile involved in the coordination of Mg^2+^ and has the consensus DxD sequence. Motif II contains a conserved serine or threonine residue, whereas motif III contains a conserved lysine residue. Motif IV typically exhibits the consensus D(X_x_)D sequence; its acidic aspartate residues and those of motif I are important to coordinate the Mg^2+^ in the active site.^45^ From these observations, we propose that the N-domain of AsDMS involving the HAD-like domain shares a common evolutionary origin with HAD-like hydrolases and acts as a HAD phosphatase by catalyzing the dephosphorylation reactions. Interestingly, the HAD motif IV conserved in AsDMS is the functional DDxxE_195–199_ motif that is also commonly found in class I terpene synthases and responsible for positioning the FPP substrate for further reactions.

Next, we traced the phylogenetic relationship of the C-domain of AsDMS with functionally characterized and unknown terpene synthases. Through gene duplication and fusion, available structures of terpene synthases contain various combinations of α, β, and γ domains: class I enzymes have α, αα, αβ, and αβγ architectures, while class II enzymes contain βγ and αβγ architectures.^10,46,47^ Given that the functional DxDTT_331–335_ motif located at the C-domain of AsDMS is also conserved among class II diterpene synthases and that class II enzymes conserve the DxDTT or DxDD motifs in a β-domain,^48,49^ we constructed the phylogeny of the C-domain of AsDMS with terpene synthase β-domains. The results demonstrate that the C-domain AsDMS was homologous to the β-domains of bacterial class II or plant terpene synthases (Figure 7B). Moreover, comparison of the amino acid alignment between the C-domain of AsDMS and β-domains of other terpene synthases indicated that the DxDTT_331–335_ motif of AsDMS corresponds closely to the DxDD or DxDTT motifs of other enzymes (Figure 8B). These findings suggest that the C-domain of AsDMS probably evolved from a class II terpene synthase β-domain.

It is interesting to note that the N-domain (HAD-like domain) and C-domain (terpene synthase β domain) of AsDMS, each evolved from different enzymes and uniquely fused (Figure 8C), substantially differentiate AsDMS from previously characterized terpene synthases.^12,35,41,46^ In light of the evolutionary history of multidomain HAD phosphatases, these enzymes have undergone remarkable expansion via gene duplication events and are accompanied by the acquisition of new domains that further diversify the protein functions.^44^ However, there are no known examples of a HAD-like domain combining with a terpene synthase domain, as observed in AsDMS. Although the evolution of the protein architecture of AsDMS remains an open question, considering that the architecture of a βγ terpene synthase arises from the fusion of the β and γ enzymes,^12^ one possibility is that AsDMS is also formed through the fusion of ancestral genes encoding discrete HAD and β proteins (Figure 8C).

### Conclusions

We identified five active DMSs of marine bacterial origin for the first time. Among the five DMSs, the *A. spongiae* AsDMS was further characterized as a bifunctional STS that utilizes two active DDxxE (N-domain) and DxDTT (C-domain) motifs for catalyzing sequential cyclization and dephosphorylation reactions to produce the final product, drimenol, from FPP. Notably, both HAD-like domain (N-domain) and terpene synthase β domain (C-domain) of AsDMS with different evolutionary origins might have become fused and formed an active site at the interface of two domains, giving rise to a unique protein architecture. Our discovery of bacterial DMSs paves the way for future studies on drimane sesquiterpenes in bacteria and highlights the diversity in the structures and functions of terpene synthases, which play increasingly significant roles in terpene biosynthesis.

## Methods

### Genomic Data Mining for Candidate Bacterial DMSs

Candidate DMSs were identified by screening all putative proteins against the HMM of AstC as a query, with HMMER web server^25^ and the NCBI database. The six selected proteins were subjected to multiple protein alignments to confirm the presence of the conserved DxDTT motif.

### Materials for Cloning and Enzymatic Activity Assays

Details are provided in Method S1.

### Plasmid Construction

The coding sequences of six candidate *DMS* genes were codon-optimized for expression in *E. coli* and synthesized by GENEWIZ (South Plainfield, NJ, USA). Each sequence was amplified by the polymerase chain reaction (PCR) using PrimeSTAR GXL DNA polymerase with gene-specific primers 1–12 (Table S1). The PCR products were purified using a FastGene gel/PCR extraction kit (Nippon Genetics, Tokyo, Japan), according to the manufacturer’s instructions, and further integrated into the linearized pET28b(+) expression vector (*Nde* I and *Xho* I) in-frame with the N-terminal His_8_-tag by In-Fusion cloning (Takara Bio, Otsu, Japan). The sequence and correct orientation of the *DMS* genes were confirmed, followed by transformation into *E. coli* BL21 Star (DE3). The empty pET28b-His_8_ vector was used as a negative control for protein expression.

Site-directed mutagenesis of *AsDMS* was performed in the pET28b-His_8_*AsDMS* clone by inverse PCR with gene-specific primers 13–24 (Table S1). Sequence determination of these mutagenized constructs was performed prior to expression.

### Expression and Purification of DMS Proteins

All recombinant N-terminal His_8_-tagged DMS proteins, including AsDMS, were expressed in *E. coli* BL21 Star (DE3) strains harboring pET28b-His_8_*DMS* and purified using TALON metal affinity resin (Clontech, Mountain View, CA, USA), as described in Method S2. The concentrations of the purified proteins were measured with a protein assay kit (Bio-Rad, Hercules, CA, USA) using bovine serum albumin as a standard. The expression and purification of AsDMS mutant proteins were performed as described for the recombinant AsDMS. These purified proteins were further used for *in vitro* enzymatic activity assays.

### *In Vitro* Enzymatic Activity Assay

For the initial characterization of the DMS proteins (WT and mutants), the reaction was initiated by the addition of purified proteins (AaDMS and AsDMS, final 200 nM; A474DMS, A119DMS, FeDMS, and RbDMS, final 500 nM) to a mixture of 50 mM Tris–HCl (pH 8.0), 2 mM MgCl_2_, 1 mM dithiothreitol, and 100 μM FPP, GPP, or GGPP. Reactions in the absence of proteins were used as negative controls. The reaction mixture (400 μL) was incubated at 30 °C for 1 h. Then, BAP from *E. coli* C75 (4 μL, 2 units; Takara Bio) was added to 200 μL of the reaction mixture and further incubated for 1 h at 37 °C. The aqueous layer was then extracted twice with 200 μL of hexane/ethyl acetate (1:1, v/v). After centrifugation at 4400*g* for 5 min (4 °C), the upper organic layer was removed, and the combined extracts were subjected to GC–MS analysis. The DMS protein assays with farnesol as a substrate (final 100 μM) were performed in the same manner as for the characterization of the DMS proteins, except for the absence of BAP treatment.

For the kinetic assay of the AsDMS (WT) protein, several reaction conditions were tested: a total volume of 200 μL of the reaction mixture containing 50 mM Tris–HCl (pH 8.0), 2 mM MgCl_2_, 1 mM dithiothreitol, 0–120 μM FPP, and 200 nM purified proteins at 30 °C within 0–15 min (during which the enzyme activity was linear). The reactions were terminated by adding 20 μL of 2.0 M HCl. A 10 μL aliquot of (+)-nootkatone (internal standard, 50 μM final concentration) was added. The products were extracted twice using 200 μL of hexane/ethyl acetate (1:1, v/v) and centrifuged at 4400*g* for 5 min (4 °C). The resulting organic layer was combined and used for the GC–MS analysis.

### GC–MS Analysis

The reaction products were detected with a 7890 GC (Agilent Technologies, Santa Clara, CA, USA) coupled with a 5975 mass selective detector and HP-5ms UI capillary column (30 m × 250 μm × 0.25 μm). A 1 μL sample was injected in splitless mode at an inlet temperature of 260 °C. The initial oven temperature of 70 °C was increased after 1 min to 230 °C at a rate of 8 °C min^–1^ and maintained for 10 min at 230 °C, followed by an increase at a rate of 20 °C min^–1^ to 300 °C. Helium was used as the carrier gas at a flow rate of 1 mL min^–1^. The amount of the reaction product was measured as the peak area using MSD ChemStation G1701EA E.02.02.1431 GC/MS software (Agilent). The results obtained from three independent experiments are expressed as means ± standard deviation (SD). The kinetic parameters were calculated by fitting a Michaelis–Menten curve to the raw kinetic data using Prism 6.0h software (GraphPad Software, Inc., San Diego, CA, USA). The raw data were calibrated in advance using a standard curve of authentic (−)-drimenol.

### Virtual Docking Studies

The three-dimensional (3D) structures of the N- and C-terminal domains of the AsDMS protein were predicted by the SWISS-MODEL^30^ pipeline using the crystal structures of the HAD-like phosphatase YihX from *E. coli* (PDB ID: 2B0C chain A)^32^ and the *ent*-copalyl diphosphate synthase PtmT2 from *S. platensis* (PDB ID: 5BP8 chain A)^33^ as template structures, respectively. These structural models were assembled using DEMO software,^34^ and a full-length model of AsDMS was obtained. The AsDMS model was also predicted using AlphaFold2.^31^ The entire docking process was performed using AutoDock Vina 1.1.2 software^37^ with the PyRx-0.8 interface.^50^ The 3D conformation of input ligand FPP was obtained from PubChem (http://pubchem.ncbi.nlm.nih.gov/), and the geometry of the FPP structure was refined to minimize its free energy state using Avogadro 1.2.0 software.^51^ The enzyme–ligand complexes with the highest docking scores acquired from the docking process were obtained, and enzyme–ligand interactions were visualized using PyMOL 1.8 (Schrödinger, LLC, New York, NY, USA). In addition, the potential conformers of FPP in the AsDMS protein, and the minimal distances between FPP and its contacting residues, were calculated using PyMOL.

### Phylogenetic Analysis

A multiple sequence alignment was generated using the GenomeNet ClustalW 1.83 server (https://www.genome.jp/tools-bin/clustalw), and the alignment was colored using the BoxShade 3.21 server (https://embnet.vital-it.ch/software/BOX_form.html). Evolutionary analyses of full-length amino acid sequences of the N- and C-terminal domains of AsDMS with other reported HAD-like hydrolases and terpene synthase β domains, respectively, were performed in MEGA 7.0,^52^ based on a neighbor-joining method using the *p*-distance algorithm with 1000 bootstrap replicates.

## Supporting Information Available

List of gene-specific primers used for PCR; expression of six recombinant DMS candidate proteins in *E. coli*; *in vitro* enzymatic assays of the recombinant AsDMS protein; *in vitro* enzymatic assays of the four recombinant proteins A474DMS, A119DMS, FeDMS, and RbDMS; *in vitro* enzymatic assays of the recombinant AaDMS protein; Michaelis–Menten curve of the AsDMS protein; Methods S1 and S2 (PDF)
