# Supplementary material for: Identification and Characterization of Bifunctional Drimenol Synthases of Marine Bacterial Origin
Source: ACS Chem Biol. 2022 Apr 21;17(5):1226–38. doi: 10.1021/acschembio.2c00163 (PMC9128629; doi:10.1021/acschembio.2c00163)
Supplement: Supplementary file 1 — cb2c00163_si_001.pdf [file cb2c00163_si_001.pdf]

Supporting Information

Identification and Characterization of Bifunctional Drimenol Synthases of Marine Bacterial Origin

Nhu Ngoc Quynh Vo,[†, *] Yuhta Nomura,[‡] Kiyomi Kinugasa,[†, §] Hiroshi Takagi,[†] and Shunji Takahashi[†, §, *]

[†]Natural Product Biosynthesis Research Unit, RIKEN Center for Sustainable Resource Science, 2–1 Hirosawa, Wako, Saitama 351–0198, Japan
[‡]Biomolecular Characterization Unit, RIKEN Center for Sustainable Resource Science, 2–1 Hirosawa, Wako, Saitama 351–0198, Japan
[§]Graduate School of Science and Engineering, Saitama University, 255 Shimo-Okubo, Sakura-ku, Saitama 338–8570, Japan

*Corresponding authors: nhu.vo@riken.jp; shunjitaka@riken.jp

Table of contents

	Page
**Table S1.** List of the gene-specific primers used for PCR in this study	S2
**Figure S1.** Expression of six recombinant DMS candidate proteins in *E. coli*.	S3
**Figure S2.** *In vitro* enzymatic assays of the recombinant AsDMS protein.	S4
**Figure S3.** *In vitro* enzymatic assays of the four recombinant proteins A474DMS, A119DMS, FeDMS, and RbDMS.	S5
**Figure S4.** *In vitro* enzymatic assays of the recombinant AaDMS protein.	S7
**Figure S5.** Michaelis-Menten curve of the AsDMS protein.	S8
**Methods S1 and S2**	S9

Table S1. List of the gene-specific primers used for PCR in this study

no.	primer name	primer sequence (5' to 3')[a]
**1**	FeDMS-forward	CCGCGCGGCAGCCATATGAAAGAACTGAGCACG
**2**	FeDMS-reverse	GTGGTGGTGCTCGAGTTAGTTGCGCTTGCTCATCAG
**3**	RbDMS-forward	CCGCGCGGCAGCCATATGAGCAGTCCACGTGTGG
**4**	RbDMS-reverse	GTGGTGGTGCTCGAGTTAGTTCATGAGATTCATGG
**5**	AaDMS-forward	CCGCGCGGCAGCCATATGAAAGAGCTGGAACTG
**6**	AaDMS-reverse	GTGGTGGTGCTCGAGTTACAGCATGGTCTCAATCG
**7**	AsDMS-forward	CCGCGCGGCAGCCATATGGATTTTAGCAGCAACG
**8**	AsDMS-reverse	GTGGTGGTGCTCGAGTTAACTCAGAATATGC
**9**	A474DMS-forward	CCGCGCGGCAGCCATATGAAGGAAGTGCTGACC
**10**	A474DMS-reverse	GTGGTGGTGCTCGAGTTAGCTCAGAATATTG
**11**	A119DMS-forward	CCGCGCGGCAGCCATATGAAAGAAGTGCTCACC
**12**	A119DMS-reverse	GTGGTGGTGCTCGAGTTAGCTCAGCAGATTCAG
**13**	AsDMS-D196N/K197D-forward	CCATCTTCATCGAC**AACGAC**AGCGAGAATCTGCAAGAGGCG
**14**	AsDMS-D196N/K197D-reverse	CGCCTCTTGCAGATTCTCGCT**GTCGTT**GTCGATGAAGATGG
**15**	AsDMS-D196N-forward	CCATCTTCATCGAC**AAC**AAGAGCGAGAATCTGCAAGAGGCG
**16**	AsDMS-D196N-reverse	CGCCTCTTGCAGATTCTCGCTCTT**GTT**GTCGATGAAGATGG
**17**	AsDMS-K197D-forward	CCATCTTCATCGACGAC**GAC**AGCGAGAATCTGCAAGAGGCG
**18**	AsDMS-K197D-reverse	CGCCTCTTGCAGATTCTCGCT**GTC**GTCGTCGATGAAGATGG
**19**	AsDMS-D195N/D196N-forward	CCATCTTCATC**AACAAC**AAGAGCGAGAATCTGCAAGAGGCG
**20**	AsDMS-D195N/D196N-reverse	CGCCTCTTGCAGATTCTCGCTCTT**GTTGTT**GATGAAGATGG
**21**	AsDMS-D195A/D196A-forward	CCATCTTCATC**GCCGCC**AAGAGCGAGAATCTGCAAGAGGCG
**22**	AsDMS-D195A/D196A-reverse	CGCCTCTTGCAGATTCTCGCTCTT**GGCGGC**GATGAAGATGG
**23**	AsDMS-D333N-forward	ATTTCCCGGACGATCTG**AAC**ACCACGAGTATGGTGCTCAGC
**24**	AsDMS-D333N-reverse	GCTGAGCACCATACTCGTGGT**GTT**CAGATCGTCCGGGAAAT

[a]The underlined sequences represent the vector sequences for In-Fusion cloning, and the bold sequences are the site-directed mutagenesis points.

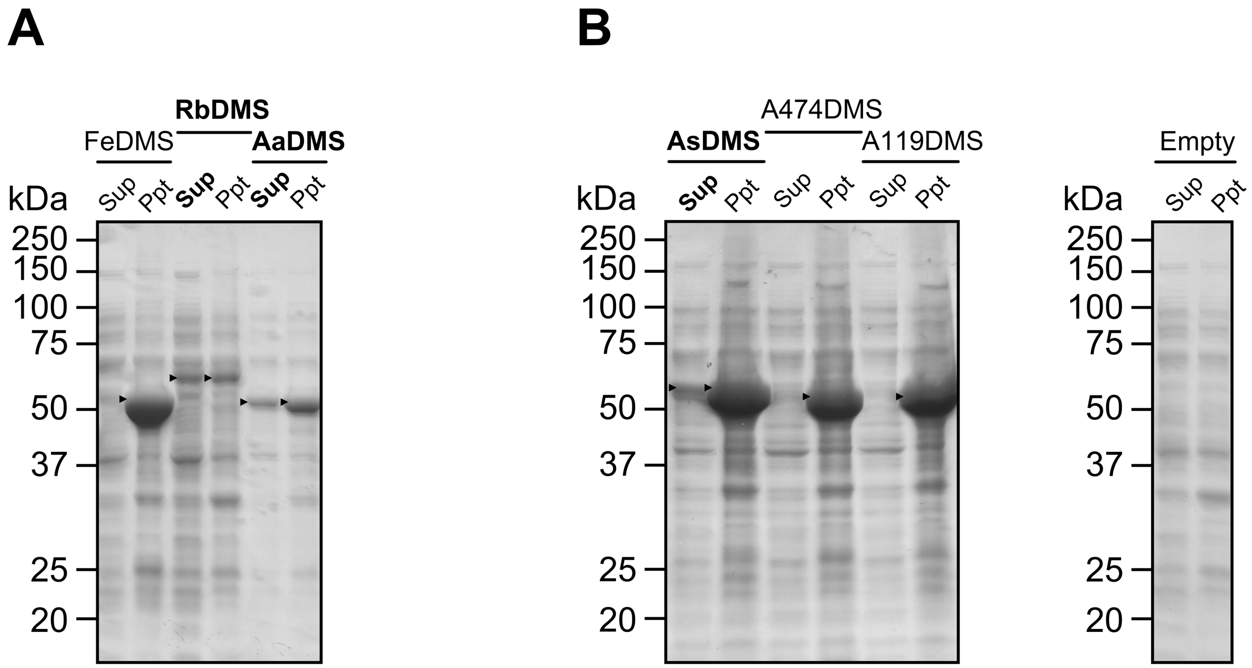

Figure S1. Expression of six recombinant DMS candidate proteins in *E. coli*. Sodium dodecyl sulfate-polyacrylamide gel electrophoresis (SDS-PAGE) analysis of (A) FeDMS, RbDMS, and AaDMS; (B) AsDMS, A474DMS, and A119DMS. The proteins were expressed and collected in the supernatant (Sup) and/or pellet (Ppt) fractions. The DMS candidates shown in bold black font were visibly expressed in soluble fractions. Expression of the empty vector was used as a negative control. The gels were stained with Coomassie Brilliant Blue, and the positions of molecular markers are indicated. *Arrowheads* indicate the DMS bands. The species names are abbreviated as follows: Fe, *Flavivirga eckloniae*; Rb, *Rhodobacteraceae* KLH11; Aa, *Aquimarina aggregate*; As, *Aquimarina spongiae*; A474, *Aquimarina* sp. AU474; and A119, *Aquimarina* sp. AU119.

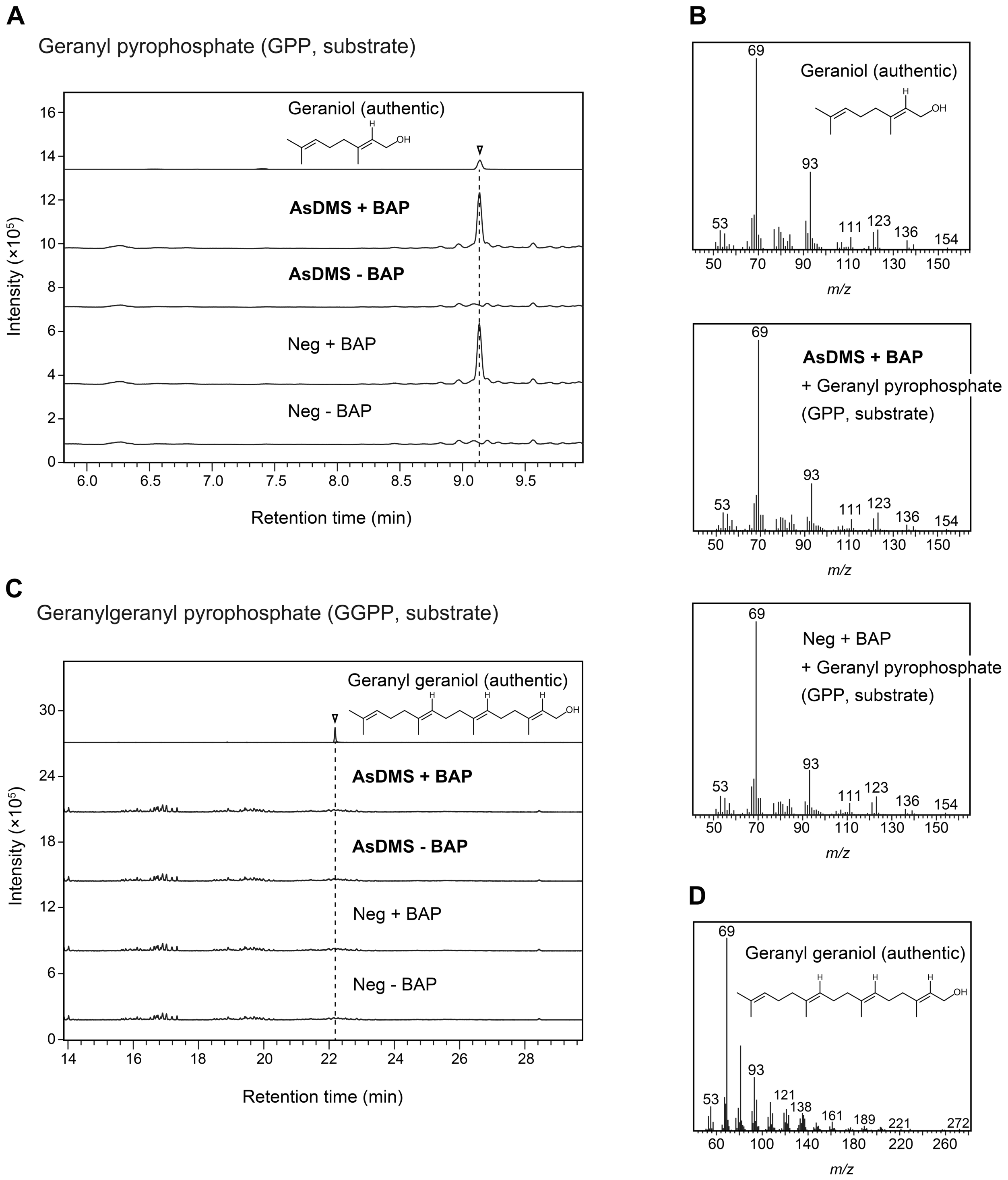

Figure S2. *In vitro* enzymatic assays of the recombinant AsDMS protein. Gas chromatography-mass spectrometry (GC-MS) analyses of the authentic compounds (geraniol and geranyl geraniol), and *in vitro* enzyme reactions of the purified AsDMS protein from *E. coli* are shown. The reactions were performed individually in the presence of geranyl pyrophosphate (GPP) (A)

or geranylgeranyl pyrophosphate (GGPP) (C) as a substrate, with or without alkaline phosphatase (BAP), as described in the Methods. The reactions in the absence of protein (Neg) were used as negative controls. (B) Mass spectra of the authentic geraniol and the reaction products detected in (A). (D) Mass spectrum of the authentic geranyl geraniol. The data in (A–D) represent three independent reactions performed with the same preparation of purified AsDMS protein. As, *Aquimarina spongiae*; and *m/z*, mass-to-charge ratio.

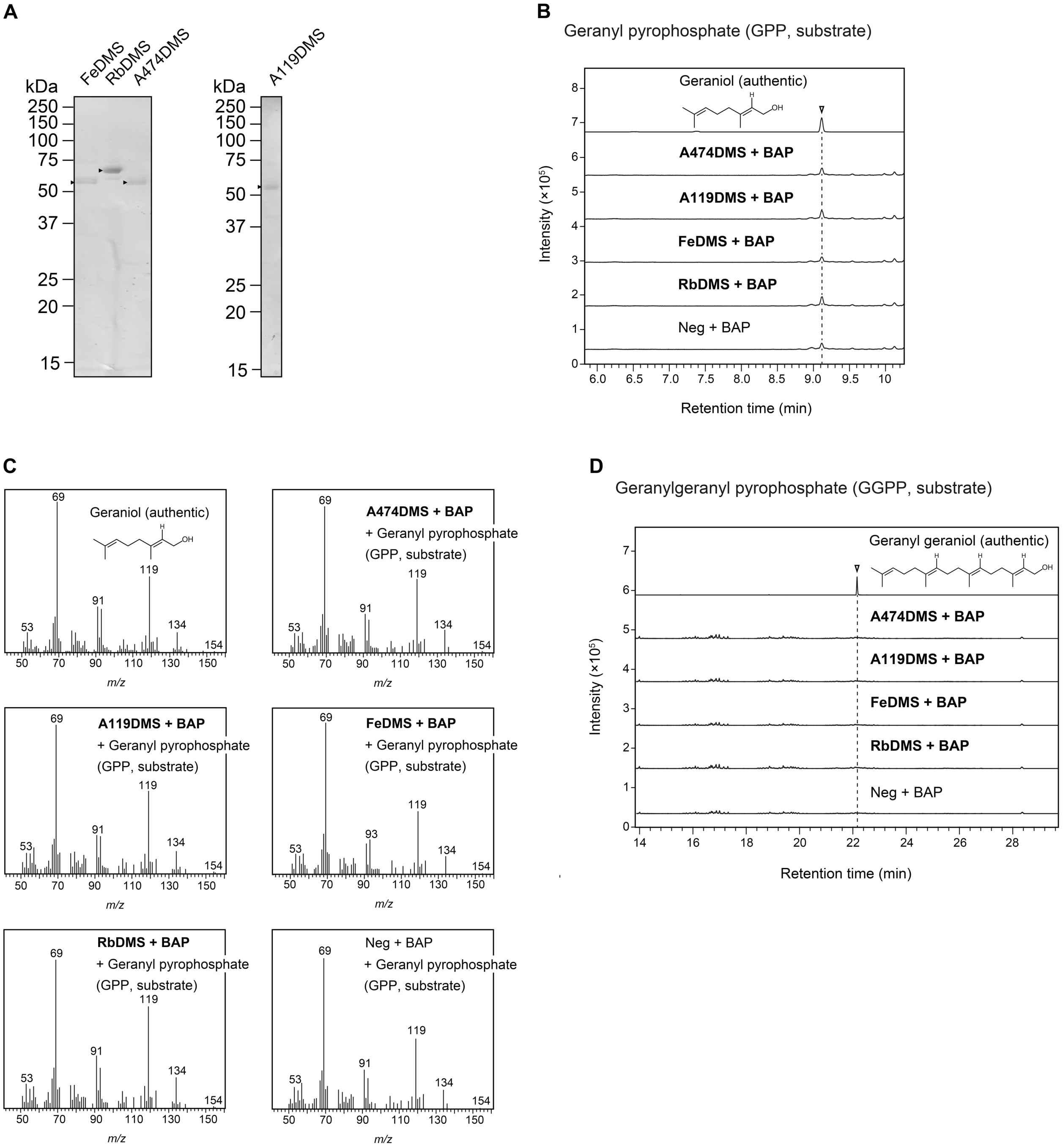

Figure S3. *In vitro* enzymatic assays of the four recombinant proteins A474DMS, A119DMS, FeDMS, and RbDMS. (A) SDS-PAGE analysis of these purified proteins from *E. coli*. The gels were stained with Coomassie Brilliant Blue, and the positions of molecular markers are indicated. The *arrowheads* indicate the DMS protein bands. (B), (D) GC-MS analyses of the authentic geraniol and geranyl geraniol, and *in vitro* enzyme reactions of the four purified proteins from *E. coli* are shown. The reactions were performed individually in the presence of geranyl pyrophosphate (GPP) (B) or geranylgeranyl pyrophosphate (GGPP) (D) as a substrate, with or without alkaline phosphatase (BAP). The reactions in the absence of protein (Neg) were used as negative controls. (C) Mass spectra of the authentic geraniol and the reaction products detected in (B). The data in (B–D) represent three independent reactions performed with the same preparation of each purified protein. A474, *Aquimarina* sp. AU474; A119, *Aquimarina* sp. AU119; Fe, *Flavivirga eckloniae*; Rb, *Rhodobacteraceae* KLH11; and *m/z*, mass-to-charge ratio.

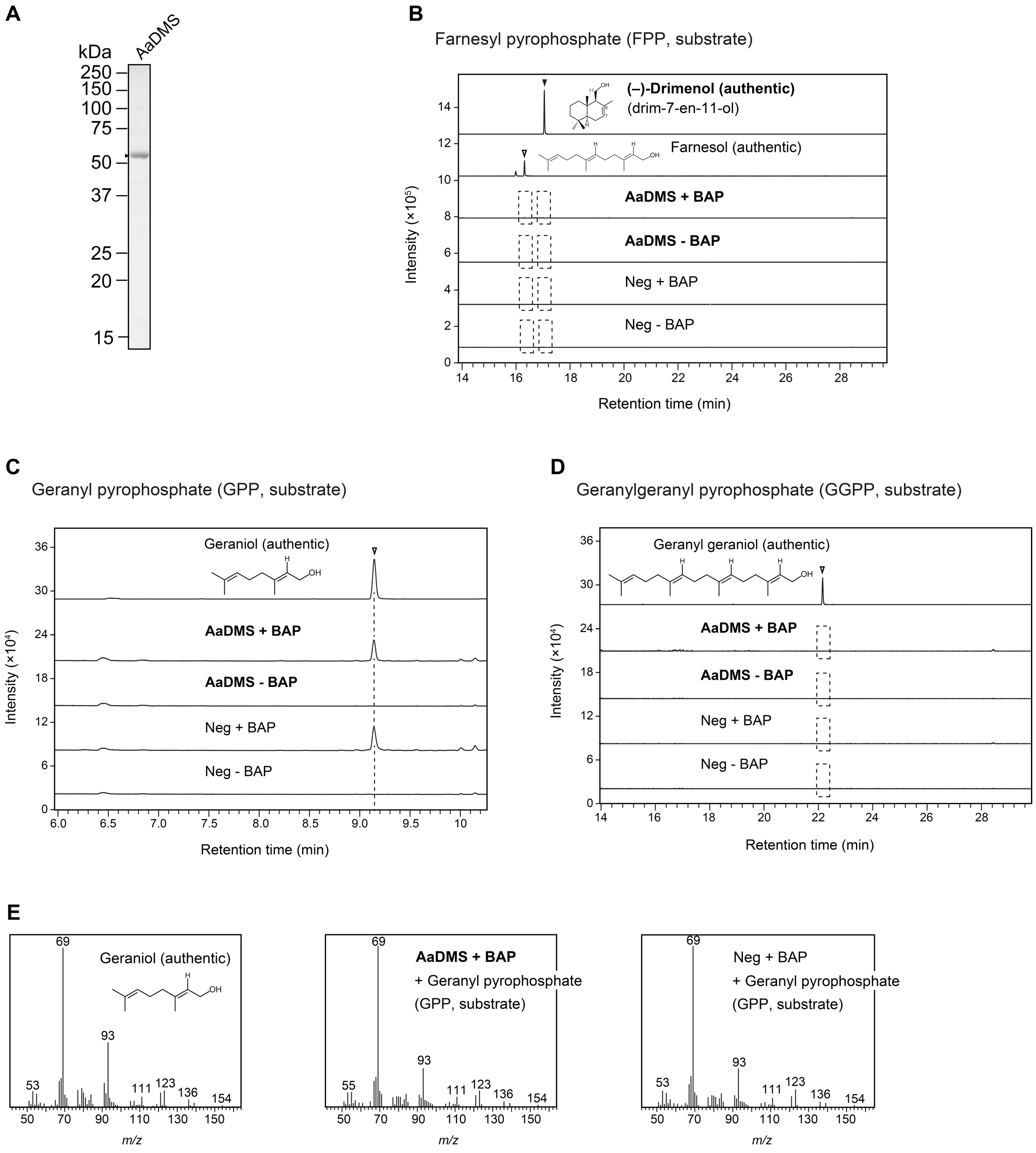

Figure S4. *In vitro* enzymatic assays of the recombinant AaDMS protein. (A) SDS-PAGE analysis of the purified AaDMS protein from *E. coli*. The gel was stained with Coomassie Brilliant Blue, and the positions of molecular markers are indicated. *Arrowheads* indicate the AaDMS band. (B–D) GC-MS chromatograms of authentic compounds (farnesol, geraniol, and geranyl geraniol), and *in vitro* reaction products are shown. The reactions were performed individually in the presence of substrates, including farnesyl pyrophosphate (FPP) (B), geranyl

pyrophosphate (GPP) (C), or geranylgeranyl pyrophosphate (GGPP) (D), with or without alkaline phosphatase (BAP). The reactions in the absence of protein (Neg) were used as negative controls. (E) Mass spectra of the authentic geraniol and the reaction products detected in (C). The data in (B–E) represent three independent reactions performed with the same preparation of purified AaDMS protein. Dashed boxes indicate that no desired reaction products were detected. Aa, *Aquimarina aggregate.*

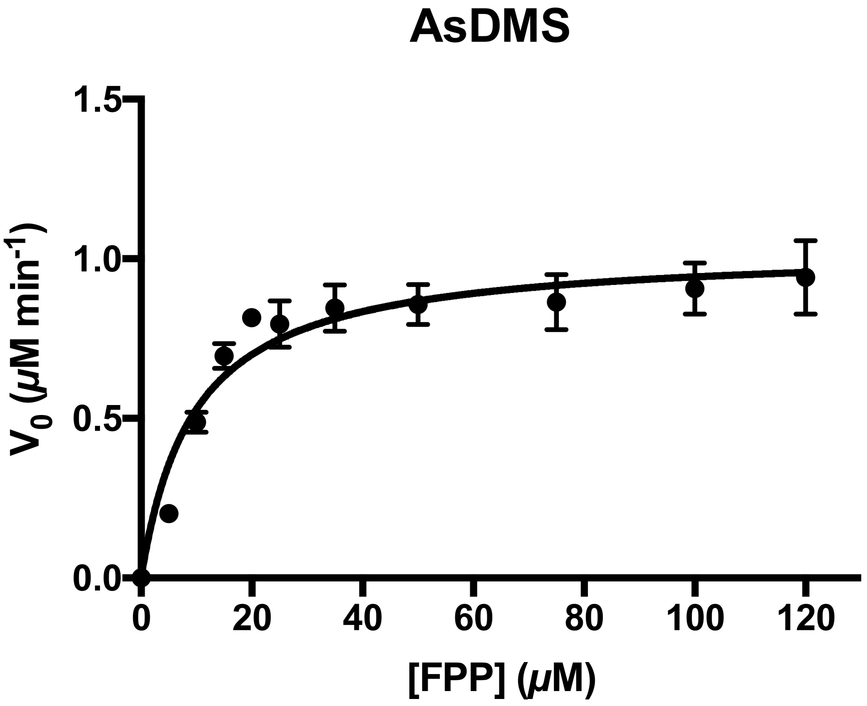

Figure S5. Michaelis-Menten curve of the AsDMS protein. The enzyme reactions of this protein were performed individually in the presence of farnesyl diphosphate (FPP) substrate, as described in the Methods. Data are the means ± SD of three separate experiments performed with the same preparation of purified AsDMS protein. As, *Aquimarina spongiae.*

Method S1. Materials for Cloning and Enzymatic Activity Assays.

Bacterial Strains and Plasmids. The *E. coli* HST08 strain was purchased from Takara Bio (Otsu, Japan). Plasmid pET28b(+) and *E. coli* BL21 Star (DE3), used for heterologous expression of the DMS proteins, were purchased from Merck Millipore (Darmstadt, Germany) and Thermo Scientific (Wilmington, DE, USA), respectively.

Chemicals and Enzymes. Substrates and authentic samples used for enzymatic assays and GC-MS analysis, including FPP, GPP, GGPP, farnesol, geraniol, and geranyl geraniol, were purchased from Sigma-Aldrich (St Louis, MO, USA). The authentic (–)-drimenol was obtained from Cayman Chemical (Ann Arbor, MI, USA). The (+)-nootkatone (Sigma-Aldrich) was used as the internal standard. Magnesium chloride and dithiothreitol were obtained from Nacalai Tesque (Kyoto, Japan). All organic solvents were of analytical grade, and were supplied by Wako Pure Chemical Industries (Osaka, Japan). Purified water was prepared using the Milli-Q purification system (Millipore, Billerica, MA, USA). Restriction enzymes used for DNA manipulations, and BAP from *E. coli* C75, were purchased from Takara Bio. PrimeSTAR GXL DNA polymerase (Takara Bio) was used in the PCR amplification. The Coomassie Brilliant Blue (EzStain Aqua) used for gel staining was purchased from ATTO (Tokyo, Japan).

Method S2

Expression and Purification of DMS Candidate Proteins. All recombinant N-terminal His_8-tagged DMS proteins were expressed in *E. coli* BL21 Star (DE3) strains harboring pET28b-His_8*DMS*. The *E. coli* strains were cultured in 50 mL of Luria-Bertani (LB) medium supplemented with 50 μg mL^{-1} kanamycin at 37 °C for 2 h until the OD_{600} had achieved 0.5. The cultures were then kept at 16 °C for 30 min, followed by supplementation of isopropyl β-D-thiogalactopyranoside at a final concentration of 500 μM, and incubated for an additional 22 h at 16 °C. The cells were harvested at 9800*g* for 5 min at 4 °C and resuspended in lysis buffer containing 50 mM sodium phosphate buffer (pH 7.3), 500 mM NaCl, and 20% glycerol. Resuspended cells were disrupted by ultrasonic treatment on ice, and the cell extracts were then centrifuged at 20400*g* for 30 min at 4 °C. The resulting supernatant fractions were bound with TALON metal affinity resin (Clontech, Mountain View, CA, USA) on a rotary shaker at 4 °C for 1 h. The resin was washed with lysis buffer supplemented with 10 mM imidazole, and recombinant His_8-tagged proteins were then eluted with the same buffer supplemented with 200 mM imidazole. The purity of the recombinant proteins was visualized by SDS-PAGE. The gels were stained using Coomassie Brilliant Blue.
